# Alternatively activated macrophages are associated with faster growth rate in vestibular schwannoma

**DOI:** 10.1093/braincomms/fcae400

**Published:** 2024-11-12

**Authors:** Grace E Gregory, Michael J Haley, Adam P Jones, Cathal J Hannan, D Gareth Evans, Andrew T King, Pawel Paszek, Omar N Pathmanaban, Kevin N Couper, David Brough

**Affiliations:** Division of Neuroscience, School of Biological Sciences, Faculty of Biology, Medicine and Health, University of Manchester, Manchester Academic Health Science Centre, Manchester M13 9PT, UK; Geoffrey Jefferson Brain Research Centre, The Manchester Academic Health Science Centre, Northern Care Alliance NHS Foundation Trust, University of Manchester, Manchester M13 9PT, UK; The Lydia Becker Institute of Immunology and Inflammation, University of Manchester, Manchester M13 9PT, UK; Geoffrey Jefferson Brain Research Centre, The Manchester Academic Health Science Centre, Northern Care Alliance NHS Foundation Trust, University of Manchester, Manchester M13 9PT, UK; The Lydia Becker Institute of Immunology and Inflammation, University of Manchester, Manchester M13 9PT, UK; Division of Immunology, Immunity to Infection and Respiratory Medicine, Faculty of Biology, Medicine and Health, University of Manchester, Manchester M13 9PT, UK; Geoffrey Jefferson Brain Research Centre, The Manchester Academic Health Science Centre, Northern Care Alliance NHS Foundation Trust, University of Manchester, Manchester M13 9PT, UK; The Lydia Becker Institute of Immunology and Inflammation, University of Manchester, Manchester M13 9PT, UK; Division of Immunology, Immunity to Infection and Respiratory Medicine, Faculty of Biology, Medicine and Health, University of Manchester, Manchester M13 9PT, UK; Geoffrey Jefferson Brain Research Centre, The Manchester Academic Health Science Centre, Northern Care Alliance NHS Foundation Trust, University of Manchester, Manchester M13 9PT, UK; The Walton Centre NHS Foundation Trust, The Walton Centre, Fazakerley, Liverpool L9 7LJ, UK; Geoffrey Jefferson Brain Research Centre, The Manchester Academic Health Science Centre, Northern Care Alliance NHS Foundation Trust, University of Manchester, Manchester M13 9PT, UK; Division of Evolution, Infection and Genomic Sciences, School of Biological Sciences, Faculty of Biology, Medicine and Health, Manchester Academic Health Science Centre, University of Manchester, Manchester M13 9PT, UK; Geoffrey Jefferson Brain Research Centre, The Manchester Academic Health Science Centre, Northern Care Alliance NHS Foundation Trust, University of Manchester, Manchester M13 9PT, UK; Division of Cardiovascular Sciences, School of Biological Sciences, Faculty of Biology, Medicine and Health, Manchester Academic Health Science Centre, University of Manchester, Manchester M13 9PT, UK; Department of Neurosurgery, Manchester Centre for Clinical Neurosciences, Salford Royal Hospital, Northern Care Alliance NHS Foundation Trust, Salford M6 8HD, UK; The Lydia Becker Institute of Immunology and Inflammation, University of Manchester, Manchester M13 9PT, UK; Division of Immunology, Immunity to Infection and Respiratory Medicine, Faculty of Biology, Medicine and Health, University of Manchester, Manchester M13 9PT, UK; Institute of Fundamental Technological Research, Polish Academy of Sciences, 02-105 Warsaw, Poland; Division of Neuroscience, School of Biological Sciences, Faculty of Biology, Medicine and Health, University of Manchester, Manchester Academic Health Science Centre, Manchester M13 9PT, UK; Geoffrey Jefferson Brain Research Centre, The Manchester Academic Health Science Centre, Northern Care Alliance NHS Foundation Trust, University of Manchester, Manchester M13 9PT, UK; The Lydia Becker Institute of Immunology and Inflammation, University of Manchester, Manchester M13 9PT, UK; Department of Neurosurgery, Manchester Centre for Clinical Neurosciences, Salford Royal Hospital, Northern Care Alliance NHS Foundation Trust, Salford M6 8HD, UK; Geoffrey Jefferson Brain Research Centre, The Manchester Academic Health Science Centre, Northern Care Alliance NHS Foundation Trust, University of Manchester, Manchester M13 9PT, UK; The Lydia Becker Institute of Immunology and Inflammation, University of Manchester, Manchester M13 9PT, UK; Division of Immunology, Immunity to Infection and Respiratory Medicine, Faculty of Biology, Medicine and Health, University of Manchester, Manchester M13 9PT, UK; Division of Neuroscience, School of Biological Sciences, Faculty of Biology, Medicine and Health, University of Manchester, Manchester Academic Health Science Centre, Manchester M13 9PT, UK; Geoffrey Jefferson Brain Research Centre, The Manchester Academic Health Science Centre, Northern Care Alliance NHS Foundation Trust, University of Manchester, Manchester M13 9PT, UK; The Lydia Becker Institute of Immunology and Inflammation, University of Manchester, Manchester M13 9PT, UK

**Keywords:** tumour-associated macrophage, inflammation, tumour micro-environment, vestibular schwannoma, acoustic neuroma

## Abstract

The variability in vestibular schwannoma growth rates greatly complicates clinical treatment. Management options are limited to radiological observation, surgery, radiotherapy and, in specific cases, bevacizumab therapy. As such, there is a pressing requirement for growth restricting drugs for vestibular schwannoma. This study explored potential predictors of vestibular schwannoma growth in depth, highlighting differences between static and growing vestibular schwannoma to identify potential therapeutic targets. High-dimensional imaging was used to characterize the tumour micro-environment of four static and five growing vestibular schwannoma (indicated by volumetric change < 20% or ≥ 20% per year, respectively). Single-cell spatial information and protein expression data from a panel of 35 tumour immune-targeted antibodies identified specific cell populations, their expression profiles and their spatial localization within the tumour micro-environment. Growing vestibular schwannoma contained significantly more proliferative and non-proliferative alternatively activated tumour-associated macrophages per millimetre square compared with static vestibular schwannoma. Furthermore, two additional proliferative cell types were identified in growing and static vestibular schwannoma: transitioning monocytes and programmed cell death ligand 1 (PD-L1+) Schwann cells. In agreement, growing vestibular schwannoma was characterized by a tumour micro-environment composed of immune-enriched, proliferative neighbourhoods, whereas static vestibular schwannoma were composed of tumour-enriched, non-proliferative neighbourhoods. Finally, classically activated macrophages significantly colocalized with alternatively activated macrophages in static vestibular schwannoma, but this sequestration was reduced in growing vestibular schwannoma. This study provides a novel, spatial characterization of the immune landscape in growing vestibular schwannoma, whilst highlighting the need for new therapeutic targets that modulate the tumour immune micro-environment.

## Teaser

Gregory *et al.* compared the tumour micro-environments of static and growing vestibular schwannoma brain tumours and identified that growing tumours had an increase in proliferative alternatively activated macrophages. Additionally, classically activated macrophages colocalized with the alternatively activated macrophages in static tumours, but this sequestration was lost in growing tumours.

## Graphical Abstract

**Graphical Abstract:**
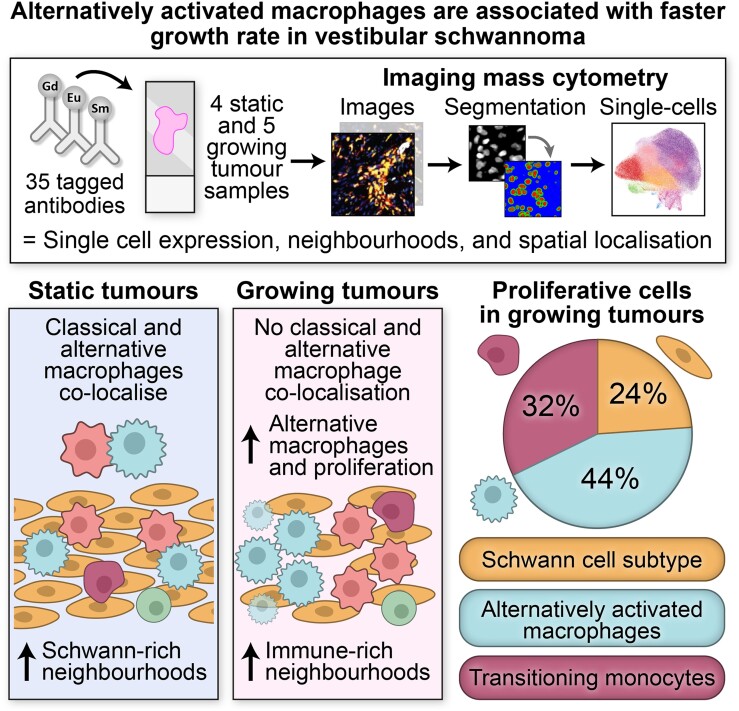

## Introduction

Vestibular schwannoma (VS), previously referred to as acoustic neuroma, are brain tumours that develop on the eighth cranial nerve in the cerebellopontine angle and within the internal auditory meatus. In 95% of patients, the VS occur unilaterally; however, in people with the rare tumour predisposition syndrome *NF2*-related schwannomatosis (*NF2*-SWN), VS arise bilaterally.^1^ Due to the close proximity of VS to the inner ear and the potential for tumour-induced mechanical pressure and inflammation of the eighth cranial nerve and brainstem, VS can cause a multitude of symptoms greatly affecting a patient’s quality of life including sensorineural hearing loss, imbalance and tinnitus.^2^ Despite a lifetime incidence of 1 in 500 people developing VS and an average volumetric growth rate of 33.5% per year, the management options available to patients are limited to radiological observation with routine MRI scans, surgical excision and radiotherapy.^1-4^ Additionally, there is only one drug option available for off-label use in VS associated with *NF2-*SWN, the anti-angiogenesis monoclonal antibody bevacizumab.^5-8^ However, in the UK, bevacizumab is not generally available for those with the more common sporadic, unilateral VS due to its side effects including serious toxicity, hypertension and impaired wound healing.^9^ Hence, new drug options are urgently required to provide alternatives for those patients with resistant or recurrent VS, and those high risk for surgical intervention.

VS exhibit heterogeneous clinical trajectories; volumetrically, the majority of tumours demonstrate growth following diagnosis, but a substantial proportion display either no clinically significant growth, or periods of growth followed by tumour stability or even shrinkage.^10,11^ Factors that correlate with growth rate include tumour-associated macrophage (TAM) infiltration and an immune-rich tumour micro-environment, although how individual cell populations contribute to tumour growth is yet to be determined.^12-14^ For example, previous studies seeking to identify a predominant population of TAMs that drive VS growth have contrastingly proposed either pro-inflammatory ‘M1 macrophages’ or anti-inflammatory ‘M2 macrophages’ dominating the tumour micro-environment.^14-16^ However, recent single-cell RNA-seq studies in VS suggest the simple M1-M2 classification may be too simplistic, with TAMs displaying a broad diversity in marker expression (such as CD163, CD68 and interleukin(IL)-1β) and behavioural plasticity, broadly encompassed by transitioning intermediate monocyte-like cells, and classically activated (formerly M1) or alternatively activated (formerly M2) states.^17-19^ Prevailing theories propose that the clinical trajectories of brain tumours are not solely influenced by the abundance of these TAMs within the tumour but rather how the TAMs compartmentalize, polarize and engage with other cells to influence cytokine or growth factor secretion and ultimately contribute to tumour progression.^17,20-22^

This study aimed to elucidate how these nuanced micro-environmental factors may correlate with VS growth rate using high-dimensional imaging to compare static and growing VS with spatial neighbourhood analysis and unsupervised clustering. By assessing specific cell types, their expression profiles, the distinct neighbourhoods they reside in and local cell–cell interactions, this study identified that significantly more alternatively activated TAMs were present in growing VS compared with static VS. In growing VS, the majority of cells were found to reside in immune-enriched, proliferative neighbourhoods within the tumour micro-environment, whilst static VSs were instead primarily composed of tumour-enriched, non-proliferative neighbourhoods. Finally, classically activated TAMs significantly colocalized with alternatively activated TAMs in static VS, but this association was lost in growing VS. By shedding light on potential cell and molecular targets within growing VS, this study provides a basis for new therapeutic targets that may be amenable to drug repurposing and immunotherapies. These data also represent a foundation for targets aimed at improving hearing outcomes in static tumours where the micro-environment is shown to be different to growing tumours.

## Materials and methods

### Sample retrieval

Four static VS (three male and one female) and five growing VS (four male and one female) cases were accessed through Health Research Authority and Health and Care Research Wales (REC reference 20/NW/0015; IRAS ID 274046; Table 1). Following clinical trial classification guidelines, static (including shrinking tumours) were defined as tumours with a preoperative growth rate of <20% volume change per year as determined by MRI volumetrics using Elements software (BrainLab), with growing tumours having a growth rate of ≥20% volume change per year.^3,23,24^ All cases were naïve from radiotherapy prior to surgery and stored as formalin-fixed paraffin-embedded blocks subsequently cut into serial 5 µm tissue faces fixed on standard slides for haematoxylin and eosin staining or for hyperion imaging mass cytometry (IMC) staining.

**Table 1:** Clinical information

Gender (age at surgery, years)	Tumour vol. (cm^3^) (months before surgery)	Growth rate (% vol. change/year)	Growth classification
F (27)	0.10 (6)	−40.00	Static
M (39)	1.66 (12)	15.79	Static
M (64)	12.4 (2)	5.91	Static
M (34)	3.54 (4)	−9.04	Static
F (43)	3.47 (10)	33.98	Growing
M (45)	2.52 (0)	170.45	Growing
M (68)	1.70 (5)	222.08	Growing
M (34)	12.1 (8)	28.02	Growing
M (62)	0.19 (2)	266.67	Growing

### Tissue staining

Standard haematoxylin and eosin-stained slides were imaged on the Olympus VS200 Slide Scanner and visualized with CaseViewer software (version 2.4, 3DHISTECH). Additionally, immunohistochemical staining for Iba1 (polyclonal rabbit anti-human, WAKO, 019-19741) was completed on serial slides of tissue to identify six Iba1+ regions of interest (ROI) per case (1060 µm^2^) as selected by a neuropathologist for IMC staining.

The 5 µm formalin-fixed paraffin-embedded tissue faces for IMC were stained in accordance with the Standard BioTools protocol.^25^ Initially, tissue was deparaffinized by 10 min submersion in xylene then 1 min each rehydration in an ethanol gradient of 100, 90, 70 and 50%, and finally ultrapure water for 5 min. Slides were incubated for 30 min at 96°C in Tris-EDTA (pH 8.5) for antigen retrieval. Slides were cooled to 70°C and washed with PBS for 8 min. Tissue was outlined with hydrophobic barrier pen and then blocked with 3% bovine serum albumin for 45 min. Biotin and then streptavidin blocks were applied for 15 min each. For PD-L1, anti-PD-L1-biotin antibody was applied for 1 h at RT and then washed, and the secondary anti-biotin antibody was incubated for 1 h at RT followed by washing. Overnight incubation occurred in a humidity chamber at 4°C with the remaining lanthanide-conjugated antibody mix diluted in PBS with 0.5% bovine serum albumin (Table 2). The 35 antibodies in the IMC panel were selected based on their established relevance in the literature for identifying cell populations (neoplastic, immune, vascular-related and proliferative cells), previous successful staining in similar Hyperion IMC studies and validation through in house optimization.^17,22^ Slides were washed twice for 8 min with 0.1% triton-100 in PBS and then PBS alone for 5 min. Cell-ID Intercalator-Iridium (Standard BioTools) was diluted at 1:400 in PBS and incubated on slides for 30 min at RT followed by a wash with ultrapure water for 10 s and dried for 20 min at RT in the dark. ROIs were then imaged on the Hyperion IMC (Standard BioTools) using laser ablation at 1 µm^2^ pixel resolution at 200 Hz frequency, from which the contents were ionized into their constituent atoms via a plasma source. The signals of the metal conjugated antibodies were identified using time-of-flight mass cytometry from within each pixel. After imaging, the images were reviewed in MCD Viewer and ROIs with unsuccessful staining were excluded from further analyses. Images were saved as 16-bit .tiff files, and ImageJ (Fiji) was used to visualize the markers of interest.

**Table 2:** Antibody details for hyperion IMC

Compartment	Acronym	Clone	Supplier	Metal tag
Neoplastic	PanCytokeratin	AE-1/AE-3	BioLegend	La139
	S100B	EP1576Y	Abcam	Pr141
	CD56	123C3	Thermo	Nd150
Immune	MHC1	EMR8-5	Abcam	Nd142
	CD14	EPR3653	Fluidigm	Nd144
	CD16	EPR16784	Fluidigm	Nd146
	CD163	EDHu-1	Fluidigm	Sm147
	CD66b	G10F5	Novus	Nd148
	CD11b	EPR1344	Fluidigm	Sm149
	Granzyme B	EPR20129-217	Abcam	Eu151
	CD45	CD45-2B11	Thermo	Sm152
	CD11c	Polyclonal	Fluidigm	Sm154
	CD4	EPR6855	Fluidigm	Gd156
	CD45RA	HI100	BioLegend	Gd158
	Iba1	Polyclonal	WAKO	Dy161
	CD8a	CD8/144B	eBioscience	Dy162
	CD74	3166025D	BioLegend	Er166
	CD3	D7A6E	Cell signalling	Er170
	CX3CR1	2A9-1	Fluidigm	Yb172
	CD45RO	UCHL1	Fluidigm	Yb173
	HLA-DR	TAL1B5	Abcam	Yb174
	CD206	E2L9N	Cell signalling	Lu175
	CD68	KP1	BioLegend	Pt198
Vascular	SMA	1A4	Bio-Rad	Y89
	CD235ab	HIR2	BioLegend	In115
	vWF	Polyclonal	Dako	Tb159
	CD31	JC/70A	Novus	Tm169
Proliferative	Ki-67	B56	Fluidigm	Er168
Other	Vimentin	RV202	Fluidigm	Nd143
	HIF-1a	EP12154	Abcam	Gd155
	VEGF	G153-694	Fluidigm	Dy163
	Fibrinogen	EPR18145-84	Abcam	Ho165
	PD-L1-Biotin (Anti-Biotin)	E1L3N (ID4.C5)	Cell signalling (BioLegend)	/Er167
	pERK1/2	D13.14.4E	Fluidigm	Yb171
	MCT4	GA5	Sigma	Yb176

### Cell segmentation

Images produced by the hyperion IMC system were denoized using IMC-Denoize to remove shot noise and hot pixels.^26^ Single-cell data were then extracted using nuclear segmentation of cells.^27^ In brief, this involved image pre-processing followed by random cropping in CellProfiler to train an object and pixel classifier for nuclei, cytoplasm and background pixels in Ilastik. Cell masks with a 1 µm expansion from the nucleus were then used to extract single-cell expression of all markers using CellProfiler. This identified a total of 223 349 cells across all the ROIs in all cases.

### Single-cell data quantification

Analysis was performed using a bespoke pipeline in Python within the Scanpy ecosystem. Mean intensities of single cells were normalized to the 99.9th percentile (scaled between 0 and 1). Cell populations were determined using unsupervised Leiden clustering, with populations being manually annotated based on known marker expression profiles, and with clusters merged if they were the same biological populations. Cell counts per square millimetre were calculated, dimensionality reduction was visualized by uniform manifold approximation and projection (UMAP), and populations were spatially mapped onto the ROIs using the locations of nuclei.

### Cell type neighbourhood analysis

To identify cell neighbourhoods, python code was adapted from Varrone *et al.*^28^ Neighbourhoods were defined at *l* = 3-step connected cells around each individual target cell, with long cell–cell links removed at the 99th percentile. Neighbourhoods were then clustered by Gaussian mixture model by their features: cell type compositions, neighbourhood shape, neighbourhood distributions and neighbourhood proximity. Fowlkes–Mallows Index identified 11 neighbourhood clusters as the most stable clustering solution, which were manually annotated into ‘tumour-enriched’, ‘vasculature-enriched’ and ‘immune-enriched’ neighbourhoods according to the dominant cell types present, and ‘proliferative’ or ‘non-proliferative’ depending on the presence or absence of proliferative cells. Neighbourhood enrichment to establish spatial proximities between neighbourhood types compared the likelihood of cells in a neighbourhood to be connected to those of a different neighbourhood against random chance (observed/expected).

### Single-cell spatial analysis

The Spatial Omics Oxford pipeline python code was adapted from Weeratunga *et al.*^29^ Quadrat correlation matrices and cross-pair correlation functions (cross-PCF) were used to identify significantly enriched spatial interactions between cell types within the ROIs. Positive or negative association was plotted across each ROI using a topographical correlation map at 100 µm. Cell types that were both significantly cooccurring by quadrat correlation matrices and significantly associated by cross-PCF were visualized in adjacency cell networks to display the proportions of interactions made by the sender to the receiver cell types, as denoted by % connections.

### Statistical analysis

Single-cell data quantification employed Shapiro–Wilk normality tests followed by two-tailed unpaired *t*-test to compare static and growing VS cell counts, and two-tailed Pearson correlations with simple linear regression against growth rate. Statistical significance when *P* < 0.05.

Cell-type neighbourhood analyses employed Shapiro–Wilk normality tests followed by either Mann–Whitney test for data without normal distribution or 2-way ANOVA for normally distributed data. Statistical significance when *P* < 0.05.

Single-cell spatial analyses employed quadrat correlation matrices to identify significant cooccurring cell types within each ROI in quadrats of 100 µm^2^ when the Benjamini–Hochberg corrected *P* value was < 0.05 with non-significant associations set to zero.^30^ For the defined significant cooccurring cell types, cross-PCF values (*g*) were established across a range of spatial distances (*r*) between the sending and receiving cells (*r* = 0–300 µm), with non-random association defined for cell types that had a *g*(*r* = 20 µm) > 1 and less interactions than would be expected by chance when *g*(*r* = 20 µm) < 1 with a 95% confidence interval after bootstrapping.^31,32^ Statistical significance when *P* < 0.05.

## Results

### Growing VS have increased myeloid and proliferation marker expression

**Figure 1:**
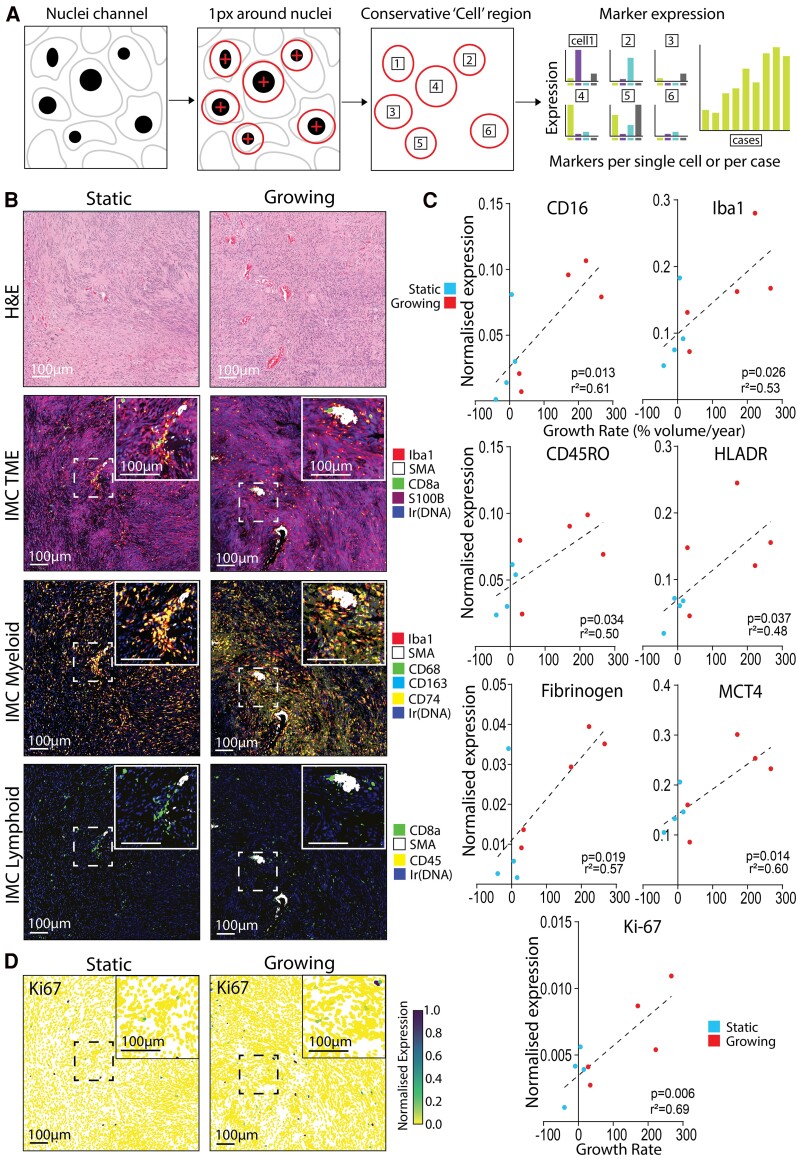
**Enriched expression of myeloid and effector T cell markers correlate with growth rate in VS.** (**A**) Schematic detailing how single cell expression data were generated from hyperion IMC images. (**B**) H&E and IMC-stained static and growing VS tissue (volume change/year < 20% or ≥ 20%, respectively) where inserts highlight areas of vascular-associated higher immune cell density within the VS region of interest. IMC images illustrate cell state–specific markers for general tumour micro-environment, myeloid cells and lymphoid cells. (**C**) Single-cell expression data extracted from the IMC images from four static (three male and one female) and five growing (four male and one female) VS and single-cell marker expression was averaged per case. All markers with expression that significantly correlated with volumetric growth rate are visualized. Shapiro–Wilk normality test followed by two-tailed Pearson correlation with simple linear regression. Correlation coefficient significance when *P* < 0.05. (**D**) Single-cell normalized expression of Ki-67, marker of cell proliferation, across all cells within the same regions of interest for static and growing VS displayed in (**B**). All scale bars denote 100 µm.

With the highly multiplexed staining of the IMC panel containing 35 antibodies, single cells from IMC images were segmented out by their nuclei, expanded to conservatively encompass cell cytoplasm and the marker expression of individual cells was calculated (Fig. 1A).

Representative images of selected antibody staining in static and growing VS are shown in (Fig. 1B), identifying Schwann (S100B), vascular (smooth muscle actin) and immune-related cells (CD68, Iba1, CD74 and CD163 for myeloid, CD45 and CD8a for T cells) within the tumour micro-environment. Single-cell marker expression data from the IMC images were averaged per case and CD16, Iba1, CD45RO, human leukocyte antigen—DR isotype, fibrinogen, MCT4 and Ki-67 significantly correlated with increasing growth rate (Fig. 1C). The increase of the proliferation marker Ki-67 with growth rate noted here was in support of previous clinical findings, as Ki-67 has often correlated with preoperative growth rate.^33^ Additionally, single-cell expression of Ki-67 (Fig. 1D) and the additional markers that correlated with growth rate (Supplementary Fig. S1) were visualized spatially matched to the ROI in Fig. 1B to display the heterogeneous nature of marker expression across the tumour micro-environment.

### Alternatively activated TAMs are more abundant in growing VS

**Figure 2:**
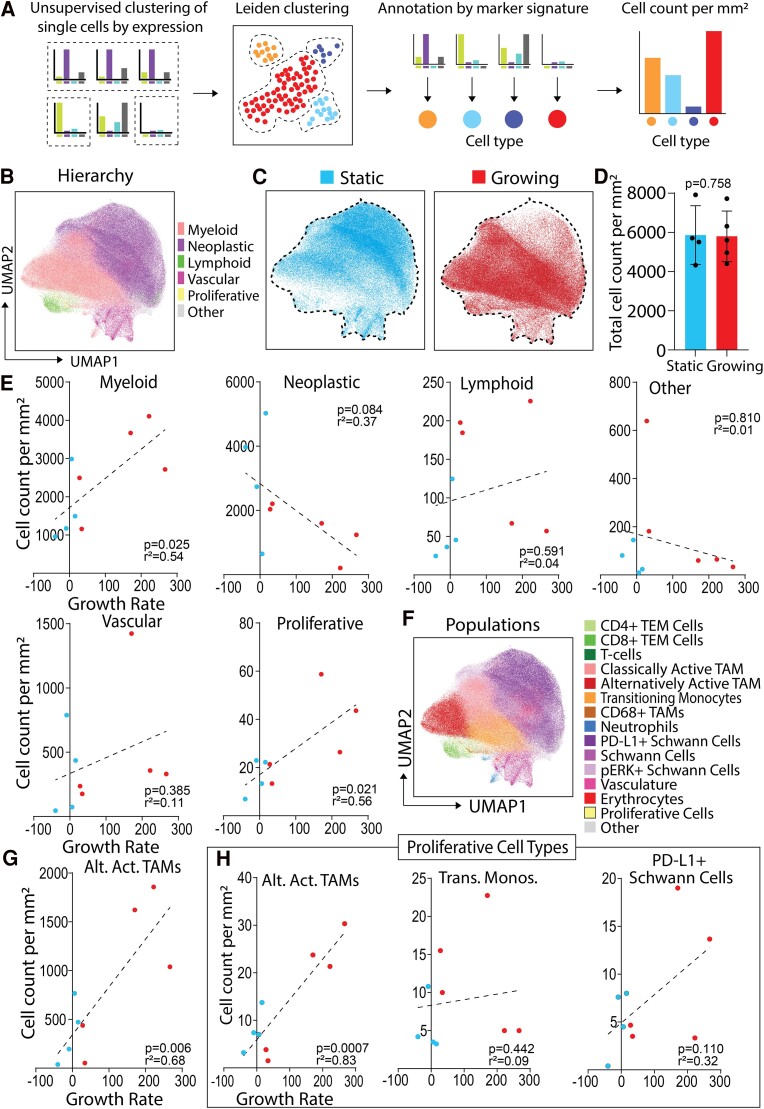
**Myeloid alternatively activated TAMs correlate with VS growth rate and have increased proliferative marker expression**. (**A**) Schematic detailing of how single-cell expression data were clustered and annotated into specific cell type populations. (**B**) Leiden clustering of all single cells from four static (three male and one female) and five growing (four male and one female) VS manually annotated into hierarchical cell types, visualized by UMAP. Static or growing VS were defined as volume change/year < 20% or ≥ 20%, respectively. (**C**) UMAPs of single cells from either static or growing VS. (**D**) Total cell count per millimetre square from static (*N* = 4) or growing (*N* = 5) cases. Shapiro–Wilk normality test followed by two-tailed unpaired *t*-test. Statistical significance when *P* < 0.05. (**E**) Correlations of cell count per millimetre square of hierarchical cell types per case (*N* = 9) with VS growth rate. Shapiro–Wilk normality test followed by two-tailed Pearson correlation or two-tailed Spearman correlation with simple linear regression for cell count per millimetre square against growth rate. Correlation coefficient significance when *P* < 0.05. (**F**) Leiden clustering and annotation of the six hierarchical cell types into 15 more granular populations, visualized on UMAP. (**G** and **H**) Statistical analysis as per (**E**). Alternatively activated TAMs correlated with volumetric growth rate (**G**). Growth rate correlations from Leiden sub-clustering of proliferative cells into proliferative populations of alternatively activated TAMs, transitioning monocytes and PD-L1+ Schwann cells (**H**). Alt. Act. TAMs, alternatively activated TAMs; trans. monos., transitioning monocytes.

As illustrated in Fig. 2A, unsupervised Leiden clustering based on single-cell marker expression was utilized to annotate cells into hierarchical cell types: ‘myeloid’, ‘neoplastic’, ‘lymphoid’, ‘vascular associated’, ‘proliferative’ or ‘other’ according to their marker expression profiles (Supplementary Fig. 2A and B), and then visualized by UMAP (Fig. 2B). This revealed good segregation between myeloid and neoplastic clusters within the UMAP space. Notably, there appeared to be a greater proportion of cells within the neoplastic cluster within the static VS cases, and a higher proportion of cells within the myeloid cluster within the growing VS cases (Fig. 2B and C). Overall, there was no significant difference in the total cell density (number of cells per square millimetre) between the two classifications (Fig. 2D). The number of myeloid cells per square millimetre was significantly positively correlated with higher growth rate (*P* = 0.025). Interestingly, no other hierarchical cell type correlated significantly with growth rate, other than the expected significant positive correlation between Ki-67+ proliferative cells and increasing VS growth rate (*P* = 0.021; Fig. 2E).

The six hierarchical cell types were further sub-classified into 15 more granular ‘populations’ annotated by specific marker expression (Supplementary Fig. S2C) and visualized by UMAP (Fig. 2F). As indicated in Supplementary Fig. S2C, Schwann cell sub-populations were S100B^high^ Cytokeratin^high^ Vimentin^high^ but were CD68^low^ CD14^low^ CD16^low^ Iba1^low^ HLADR^low^, which were instead expressed by myeloid populations. Specific myeloid sub-populations varied in pan-macrophage marker expression of CD68 and Iba1, but distinct populations like alternatively activated TAMs were CD16^high^ CX3CR1^high^ CD163+ CD11b+ CD56− and classically activated TAMs were CD16^low^ CX3CR1^low^ CD163− CD11b− CD56 + . All population cell counts against growth rate are depicted in Supplementary Fig. S3. Interestingly, only alternatively activated TAMs (and the proliferative group noted previously in Fig. 2E) significantly correlated with growth rate (*P* = 0.006; Fig. 2G). Therefore, alternatively activated TAMs represented the key myeloid populations that were significantly increased in growing VS.

Further, Leiden sub-clustering on the ‘proliferative’ cells revealed three distinct proliferative populations annotated according to their marker expression profiles: proliferative alternatively activated TAMs, proliferative transitioning monocytes and proliferative PD-L1+ Schwann cells (Supplementary Fig. S4A). Alternatively activated TAMs were the most abundant proliferative cell population in both static and growing VS (Supplementary Fig. S4B) and were the only proliferative cell type that significantly correlated (*P* = 0.0007) with growth rate (Fig. 2H). Additionally, the expression profiles of these proliferative alternatively activated TAMs were highly similar in static and growing VS, as only one of the markers in the IMC panel (MCT4) was significantly correlated with growth rate (Supplementary Fig. S4C).

### Growing VSs are composed of immune-enriched cell neighbourhoods

**Figure 3:**
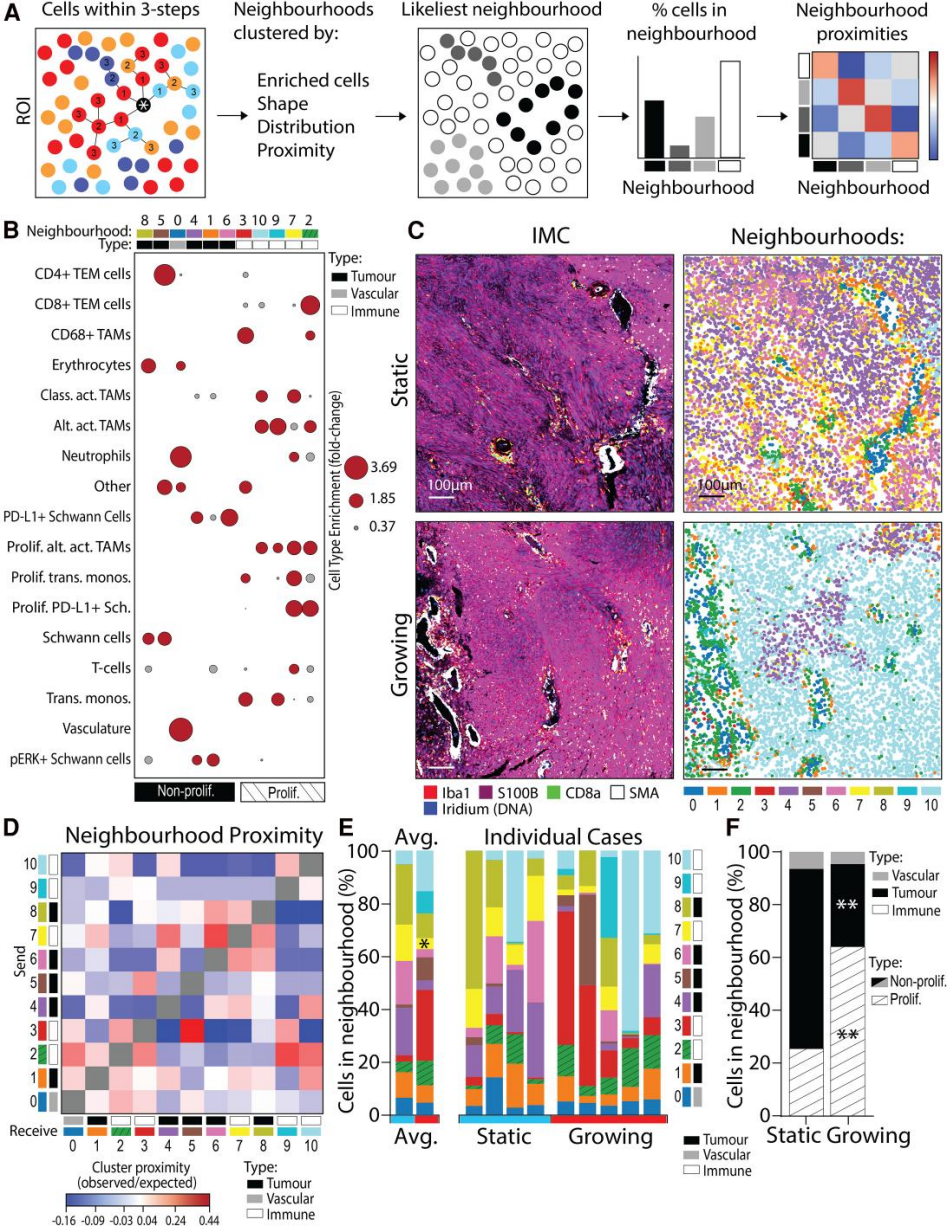
**Growing VS have more cells residing in proliferative, immune-enriched neighbourhoods**. (**A**) Schematic detailing how the cells within a three-step connection from the target cell define a single cell’s local environment (neighbourhood). (**B**) Cell types enriched within each neighbourhood plotted and used to annotate whether neighbourhoods were tumour-enriched, vasculature-enriched or immune-enriched, as well as whether they contained proliferative cells. Fold change (FC) of cell type enrichment represented by node size, with grey FC < 1 and red FC ≥ 1. Neighbourhood cell type enrichment analysis included all cells from all VS cases (*N* = 9). (**C**) IMC images with matched neighbourhood plots of cells coloured by 11 annotated neighbourhoods. Scale bars denote 100 µm. (**D**) Neighbourhood proximity analysis used pairwise analytical neighbourhood enrichment (CellCharter) to define whether pairs of the 11 neighbourhoods were likely to associate positively or negatively with one-another when compared with random chance (observed/expected). Neighbourhood proximity analysis included all cells from all VS cases (*N* = 9). (**E**) Proportions of cells within each neighbourhood by individual case and averaged from in four static (three male and one female) and five growing (four male and one female) VS. Static or growing VS defined as volume change/year < 20% or ≥ 20%, respectively. Shapiro–Wilk normality test followed by Mann–Whitney test for static (*N* = 4) and growing (*N* = 5) cases. (**F**) Proportion of cells in proliferative/non-proliferative neighbourhoods in static and growing VS, as per annotation in (**D**). Shapiro–Wilk normality test followed by 2-way ANOVA for static (*N* = 4) and growing (*N* = 5) cases. Statistical significance *P < 0.05, **P < 0.01. Prolif., proliferative; Alt. Act. TAMs, alternatively activated TAMs; Class. Act. TAMs, classically activated TAMs; trans. monos., transitioning monocytes; IMC, imaging mass cytometry.

Cell neighbourhood analysis approaches have been successfully applied to disease tissue, including cancer, to establish how cell arrangement relates to clinical outcomes, such as poorer prognosis.^28^ This study applied methodology laid out in Varrone *et al.* (2024) to investigate the cellular neighbourhoods of static and growing VS, schematically summarized in Fig. 3A. Eleven neighbourhoods were identified and annotated according to the types of cells they contained (Fig. 3B). This resulted in one vascular-enriched neighbourhood (containing erythrocytes, neutrophils and vasculature-associated cells), five tumour-enriched neighbourhoods (with Schwann cells, PD-L1+ Schwann cells and pERK+ Schwann cells), and five immune-enriched neighbourhoods [dominant in TAMs, effector memory T cells (TEM), monocytes and neutrophils]. Neighbourhoods were also annotated according to whether they contained proliferative cells, resulting in six non-proliferative and five proliferative neighbourhoods. Interestingly, none of the vascular- or tumour-enriched neighbourhoods contained proliferative cells, whereas all the immune-enriched neighbourhoods contained proliferative cells.

As shown in Fig. 3C, neighbourhoods were visualized per ROI and displayed key overlapping niches with the original IMC images. For example, the vascular-enriched neighbourhood 0 was localized to regions of smooth muscle actin staining in the IMC images, and immune-enriched neighbourhood 2 (containing TAMs and TEMs) overlapped with regions of strong Iba1+ CD8 + staining. Figure 3D displays relative neighbourhood cluster proximity (observed/expected) to indicate the potential association of neighbourhood pairs when compared against random distribution. Interestingly, the top 3 neighbourhoods with the strongest spatial associations were immune-enriched neighbourhoods (3, 2 and 7) associating with neighbourhoods 5 (tumour-enriched), 9 (immune-enriched) and 6 (tumour-enriched), respectively (Fig. 3D). Four of the neighbourhoods that contained proliferative cell types (2 containing all proliferative cell types, 3 containing proliferative transitioning monocytes and 9 and 10 containing alternatively activated TAMs) had increased pairwise spatial colocalization together within VS. However, proliferative neighbourhood 7 (strongly enriched in all three proliferative cell types) did not have positive spatial colocalization with these other proliferative neighbourhoods and instead associated with tumour-enriched neighbourhoods (1, 4, 6 and 8).

The averaged and case-by-case breakdown of the proportion of neighbourhoods shown in Fig. 3E for static and growing VS indicated a significant increase in cells residing in proliferative neighbourhood 3 (containing CD68+ TAMs, proliferative and non-proliferative transitioning monocytes and ‘other’ cells) in growing VS (*P* = 0.03). However, there was also a significant decrease (*P* = 0.03) in growing VS of cells residing in proliferative neighbourhood 7 (containing classically activated TAMs, alternatively activated TAMs, neutrophils, T cells and all proliferative cell types).

Interestingly, static cases had a significant increase in cells residing in tumour-enriched neighbourhoods (*P* = 0.0049; Fig. 3F). However, growing cases had a significant increase in cells residing in immune-enriched neighbourhoods (*P* = 0.0036). Additionally, the majority (64%) of all the cells in growing VS was found in proliferative neighbourhoods compared with only 25% of the cells in static VS (*P* = 0.016). Therefore, there was an increase in cells residing in proliferative neighbourhoods in growing VS.

### Classically activated TAMs associate with alternatively activated TAMs in static but not growing VS

**Figure 4:**
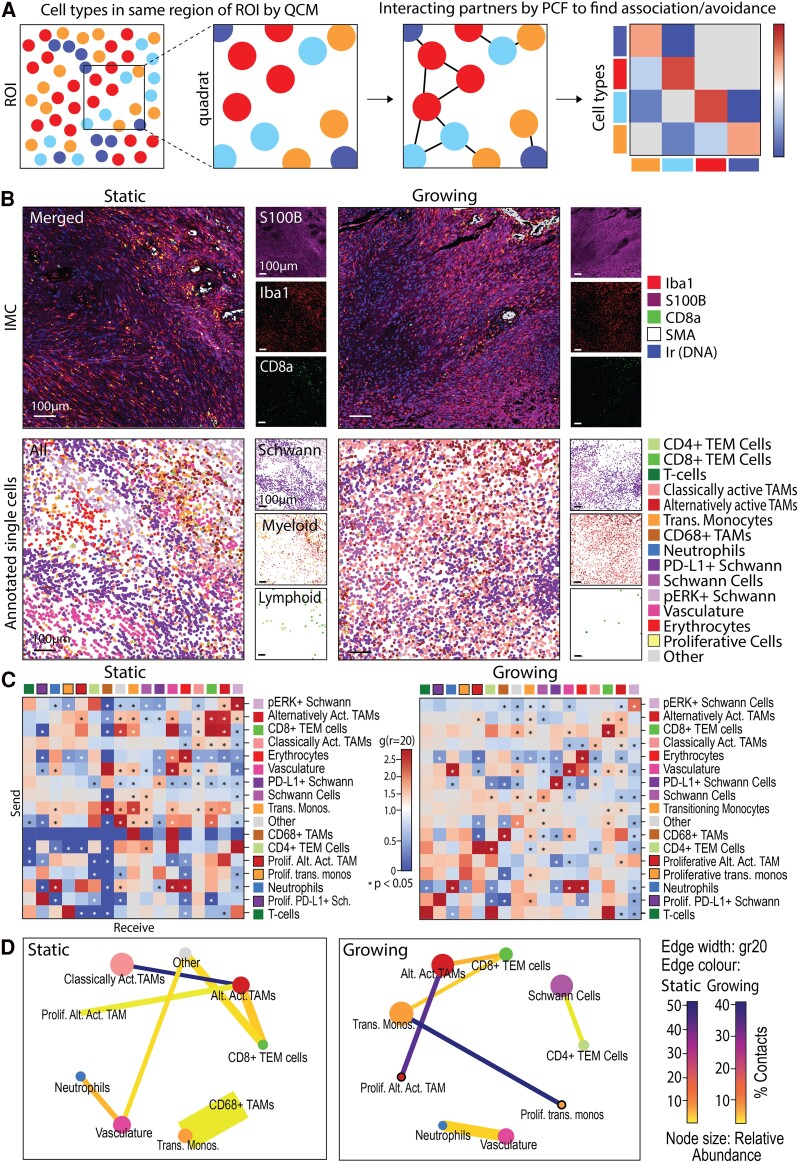
**Classically activated TAMs associate with alternatively activated TAMs in static but not growing VS**. **(A)** Schematic detailing how significant positive and negative cell–cell spatial associations are first determined by QCM in 100 µm^2^ quadrats and then cross pair correlation functions (cross-PCF). (**B**) IMC images matched with centroid XY location plots of cells coloured by their annotated populations. Scale bars denote 100 µm. (**C**) Significant positively and negatively associating cell type pairs at a distance of 20 µm (gr20) by cross-PCF (significance when *P* < 0.05) in static (*N* = 4, three male and one female) and growing (*N* = 5, four male and one female) VS cases (volume change/year < 20% or ≥ 20%, respectively). (**D**) Summary adjacency cell networks of cell populations in static (*N* = 4) and growing (*N* = 5) VS indicating significant spatial associations by both QCM and PCF (significance when *P* < 0.05). Edge width proportional to gr20, edge colour by % total contacts of the sending cell type to receiving cell type. Node size by proportional relative abundance, node colour by cell type. prolif., proliferative; trans. monos., transitioning monocytes; Sch., Schwann cells; Alt. Act. TAMs, alternatively activated TAMs.

Whilst there was an increase in proliferative cells in growing VS, the neighbourhood analyses revealed their localization with other cell types differs with growth rate, such as with classically activated TAMs, neutrophils and T cells. Therefore, specific cell–cell colocalization at the single-cell level was assessed in static and growing VS tumours to investigate whether the localizations identified in the different neighbourhoods may influence the tumour micro-environment through direct cell–cell associations. Briefly, significant cooccurrences of population pairs within 100 µm^2^ quadrats of the ROIs were identified, followed by the identification of significant positive and negative cell–cell associations (defined as a distance of 20 µm; Fig. 4A).

Identified cell populations were spatially mapped within the ROI by their centroid X and Y locations, visually giving more granular context to the IMC images of the tumour micro-environment (Fig. 4B). The significant pairwise interactions by PCF of these populations indicated positive or negative association between individual cell types within the tumour micro-environment. A difference in the spatial micro-environment organization between different cell populations in the static and growing VS was noted. For example, alternatively activated TAMs significantly positively or negatively associated with 11 cell types within static VS, but only six in the growing VS. Overall, a greater organization in static VS was suggested by the increased number of significant positively and negatively associating populations when compared with growing tumours (Fig. 4C).

Similarities in cell–cell associations were noted in static and growing VS. For example, an enriched association of alternatively activated TAMs and CD8+ TEM cells was observed in both static and growing VS, as found in the immune-enriched neighbourhood 2 in Fig. 3B. However, the CD8+ TEM cells in growing VS also showed significant association with transitioning monocytes not seen in static VS, thus indicating strong CD8+ TEM-myeloid interactions in growing VS. Additionally, although found in a higher abundance in the growing VS, proliferative alternatively activated TAMs associated with non-proliferative alternatively activated TAMs in both static and growing VS (Fig. 4C and D).

There were also significant differences in cell–cell associations between static and growing VS. Interestingly, classically activated macrophages were significantly associated with alternatively activated macrophages in static VS, but this association was not significant in growing VS (Fig. 4D). This indicated the sequestration of alternatively activated TAMs by classically activated TAMs in static VS may have been reduced in growing VS. The significant association between classically activated and alternatively activated TAMs in static VS was visualized for each case in Supplementary Fig. S5, with local clustering heatmaps highlighting regions of organization and overlap in classically activated and alternatively activated TAM location within the tumour tissue. In growing VS, regions of negative associations were noted where there was a reduction in the overlap of classically activated and alternatively activated TAM clustering (Supplementary Fig. S5). Together, these data show that classically activated TAMs associate with alternatively activated TAMs in static but not growing VS, indicating differences in the spatial immune landscape may contribute to VS growth.

## Discussion

This study aimed to address the spatial immunological determinants of VS growth. By investigating nuanced differences between VS of differing growth rates, this study identified several micro-environmental differences between static and growing tumours that may synergistically promote tumour progression within the tumour immune micro-environment of VS.

This study observed a marked increase in myeloid cells in growing VS and a shift from a neoplastic Schwann cell-rich phenotype in the static VS to an immune-rich phenotype in growing tumours. A significant increase in myeloid cells in growing VS was confirmed, which was specifically attributed to both proliferative and non-proliferative alternatively activated TAMs. Whilst Jones *et al.*^17^ indicated that alternatively activated macrophages are less abundant than classically activated TAMs in *NF2*-SWN VS, several studies indicate an important role for alternatively activated TAMs in VS progression. Alternatively activated TAMs contribute to solid tumour growth due to their role in growth factor secretion and anti-inflammatory cytokine release.^20^ However, alternatively activated TAMs also display plasticity in VS, and Baruah *et al.*^19^ identified a CD163+ TAM population that also express pro-inflammatory cytokine signatures such as IL-1β. Overall, in VS, an increase in alternatively activated TAM density has previously been associated with increased growth, shorter progression-free survival and poorer hearing outcomes.^12,14,34,35^

Alternatively activated TAMs were the most abundant proliferative cell type in both static and growing VS. Furthermore, a significant positive correlation between proliferative alternatively activated TAMs was identified in the growing VS. These data indicated ‘correlation’ of alternatively activated TAMs with VS growth rate, rather than ‘causation’. Therefore, further studies are required to elucidate the actions of these TAMs in the VS tumour micro-environment, such as *in vivo* testing of therapeutic interventions that specifically modulate alternatively activated TAMs. An example of this includes the anti-IL-4Rα monoclonal antibody dupilimab used in conjunction with PD-1/PD-L1 checkpoint blockade, which is a strategy being investigated in other cancers (e.g. non–small cell lung cancer ClinicalTrials.gov identifier NCT05013450).^20,36^ This course of combination therapy could potentially be effective in VS, as proliferative PD-L1+ Schwann cells were also noted in the present study. The strong expression of PD-L1 in this proliferative neoplastic population was suggestive of a selection bias for PD-L1+ Schwann cells that are able to evade cytotoxic T cells. Mechanistically, the binding of PD-L1 with PD-1 between tumour cells and T cells promotes a pro-tumourigenic and immunosuppressive environment within tumours and reaffirms the PD-1/PD-L1 axis for immunotherapeutic targeting to modulate the immune tumour micro-environment in VS.^17,34,37-40^ Promisingly, there has been a single case report to date demonstrating an apparent response to immune checkpoint inhibition in VS.^40^

Interestingly, proliferative alternatively activated TAMs expressed more MCT4 in growing VS. The role of MCT4 as a lactate and H^+^ export pump causes extracellular acidification, a key characteristic of fast growth and therapeutic resistance in a variety of solid tumours and found to correlate with VS tumour size.^19,41,42^ This may implicate MCT4 and other monocarboxylate transporters such as MCT1 as previously unidentified therapeutic avenues in VS. Syrosingopine, a well-tolerated approved antihypertensive drug, is both an MCT4 and MCT1 inhibitor. Syrosingopine acts synergistically in combination with the established type 2 diabetes drug metformin to reduce cancer cell viability *in vitro* in primary and immortalized cancer cell lines, as well as *in vivo* in a murine model of liver cancer.^43,44^ Applications of syrosingopine in preclinical VS models should be explored to investigate its effects on tumour acidification, immune cell proliferation and tumour growth.

The cell–cell proximity analysis of the VS tumour micro-environment revealed a significant association of classically activated TAMs with alternatively activated TAMs in static VS, which was subsequently lost in growing VS. Additionally, in growing VS, there was a significant decrease in proliferative neighbourhood 7 where classically activated TAMs and alternatively activated TAMs coexisted. When classically and alternatively activated TAMs are closely colocalized in static VS, their opposing pro- and anti-inflammatory cytokine release, competition for arginine by nitric oxide synthase and arginase-1 expression, and direct receptor-ligand interactions may reduce the tumourigenic activity of alternatively activated TAMs.^45^ However, environments that support alternatively activated macrophages can prevent classically activated macrophage function, which may be a contributing factor towards the loss of interaction between these TAM sub-types in growing VS.^46^ A loss of this interaction may allow for increased proliferation and secretion of growth factors. As such, a reduction in classically activated TAM-driven sequestration may indirectly enable proliferative neighbourhoods to become established in growing VS. The significant increase in cells residing in immune-enriched/proliferative neighbourhoods in growing VS indicates a potential for therapeutic targets that modulate growing VS neighbourhoods. For example, rebalancing the distribution of cells in growing VS towards non-proliferative tumour- and vasculature-enriched neighbourhoods may emulate a more static VS tumour micro-environment and therefore reduce tumour growth rate. In previous imaging and histopathology studies in VS, it has been suggested that vascular normalization (such as that perceived after treatment with the antiangiogenic agent bevacizumab) may have the added benefit of reducing TAM infiltration, which, therefore, could reduce the number of cells in immune-rich/proliferative neighbourhoods noted in the present study.^47,48^

Our study also highlights a potentially important limitation in the ‘static’ and ‘growing’ nomenclature often used in clinical practice and as cut-offs for clinical trial inclusion. VS are often dichotomized into ‘static’ or ‘growing’ (with linear measurements <2 mm or >2 mm or tumour volume change per year < 20% or ≥ 20%, respectively), but this may not directly translate to dichotomized biological readouts, which may follow more of a variable scale. Additionally, variability in the time period between the final MRI scan a patient receives and the date of surgery may limit the accuracy of strict ‘static’ and ‘growing’ growth classifications, as in this study this interval ranges from 0 to 12 months. Often in this study when correlating biological findings (such as the number of alternatively activated TAMs) against specific volumetric growth rate, the ‘static’ samples (that ranged between −40% and +16% annual growth rate) clustered together with the slower yet still ‘growing’ VS (+28% to +34%) that were close to the threshold for measurement error in MRI scans.^49^ However, the three much faster growing VS samples (+170% to +267%) often showed markedly different biological behaviour, for example, displaying dramatically increased alternatively activated macrophage and proliferative cell count per millimetre square (Fig. 2G). This may affect clinical decision-making in the future as patients with very rapidly growing VS may benefit from different treatments compared with those that are closer to 20% volume change/year. For example, as VS display high numbers of classically activated TAMs, static VS may benefit more from therapeutics targeted towards pro-inflammatory cytokines, such as anakinra or canakinumab for targeting IL-1β to prevent hearing loss.^17,21^ In addition to growth rate–specific clinical management, clinical trials for VS may also benefit by reformatting into growth rate–specific sub-groups, which may affect dosing, treatment regimens and have different primary outcomes.

Finally, although this study focussed on sporadic VS, treatments are also urgently needed for *NF2*-SWN patients with VS. Recent studies investigating the similarities in the tumour micro-environments of *NF2*-SWN and sporadic VS indicate that the present findings and clinical implications are also likely to be applicable to those with *NF2*-SWN VS.^47,50^ Rapid translation of therapeutics to the clinic can be achieved on a compassionate use basis for *NF2*-SWN VS, as was the case for bevacizumab due to the severity of the *NF2*-SWN disease.^51^ Therefore, this study provides a foundation for the mechanisms of growth in sporadic VS that may prove to be equally valuable therapeutic targets in *NF2*-SWN VS with faster translational potential.

## Conclusion

This study highlights the significance of alternatively activated TAMs in growing VS, characterized by their proliferative nature, correlation with growth rate, and expression profiles linked to tumour micro-environment acidification. The distinct micro-environmental characteristics of static and growing tumours may offer viable therapeutic avenues for further investigation in both sporadic and *NF2*-SWN VS.

## Supplementary Material

fcae400_Supplementary_Data

## Data Availability

The data that support the findings of this study are openly available in a FigShare repository at http://doi.org/10.48420/26510758. No novel code was generated for this study as all data were analysed using public python packages and workflows referenced in the Methods section.
