# Supplementary material for: Alternatively activated macrophages are associated with faster growth rate in vestibular schwannoma
Source: Brain Commun. 2024 Nov 12;6(6):fcae400. doi: 10.1093/braincomms/fcae400 (PMC11604085; doi:10.1093/braincomms/fcae400)
Supplement: fcae400_Supplementary_Data [file fcae400_supplementary_data.pdf]

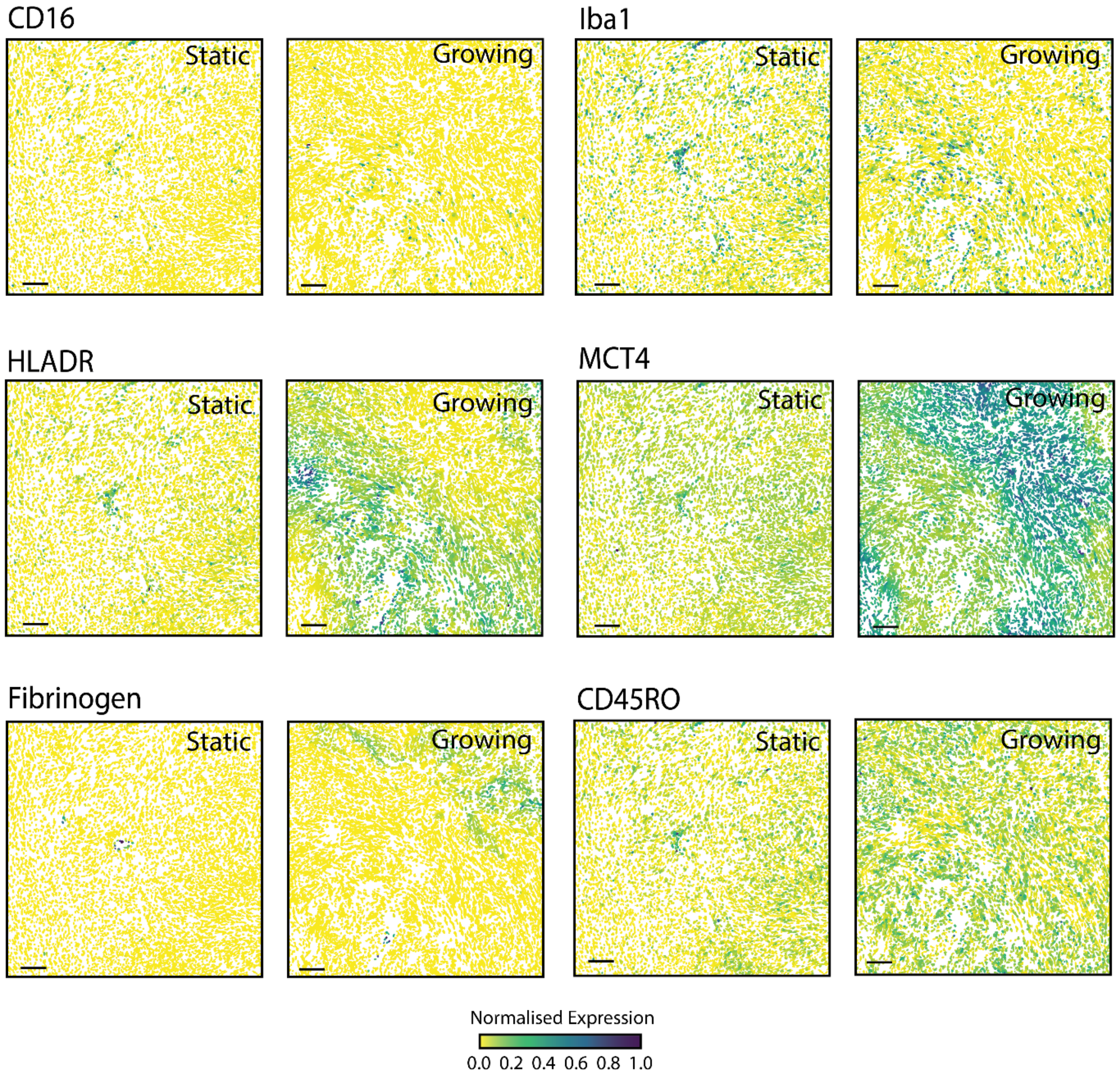

Supplementary Figure 1. Spatial maps of marker expression in all single cells of key markers significantly correlated with faster growing VS. Marker expression overlayed on single cell masks for CD16, Iba1, HLADR, MCT4, fibrinogen, and CD45RO. Representative cases for static and growing vestibular schwannoma (VS) samples from original cohort of 4 static (3 male, 1 female) and 5 growing (4 male, 1 female) patients. Static or growing VS were defined as volume change/year < 20% or ≥ 20%, respectively. Scale bars denote 100 µm.

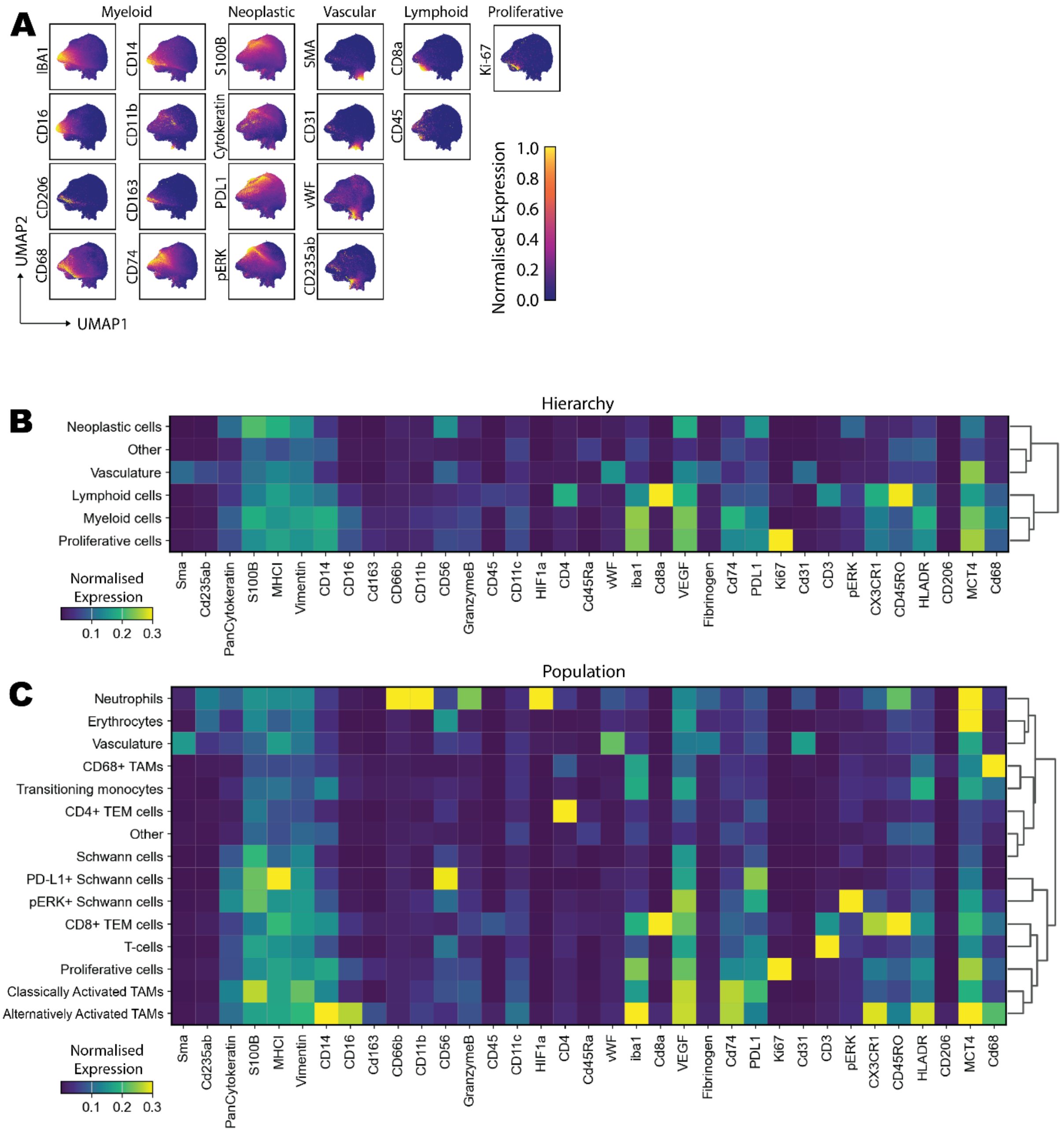

Supplementary Figure 2. Marker expression UMAPs and heatmaps of hierarchical and population cell types. (A) Single cell marker expression visualised by UMAP used to manually annotate hierarchical groups. **(B)** Heat mapped expression of imaging mass cytometry (IMC) panel markers as per hierarchy cell type from all VS cases (N=9). **(C)** Heat mapped expression of IMC panel markers as per population cell type from all VS cases (N=9). Abbreviations: effector memory T cell (TEM), vestibular schwannoma (VS).

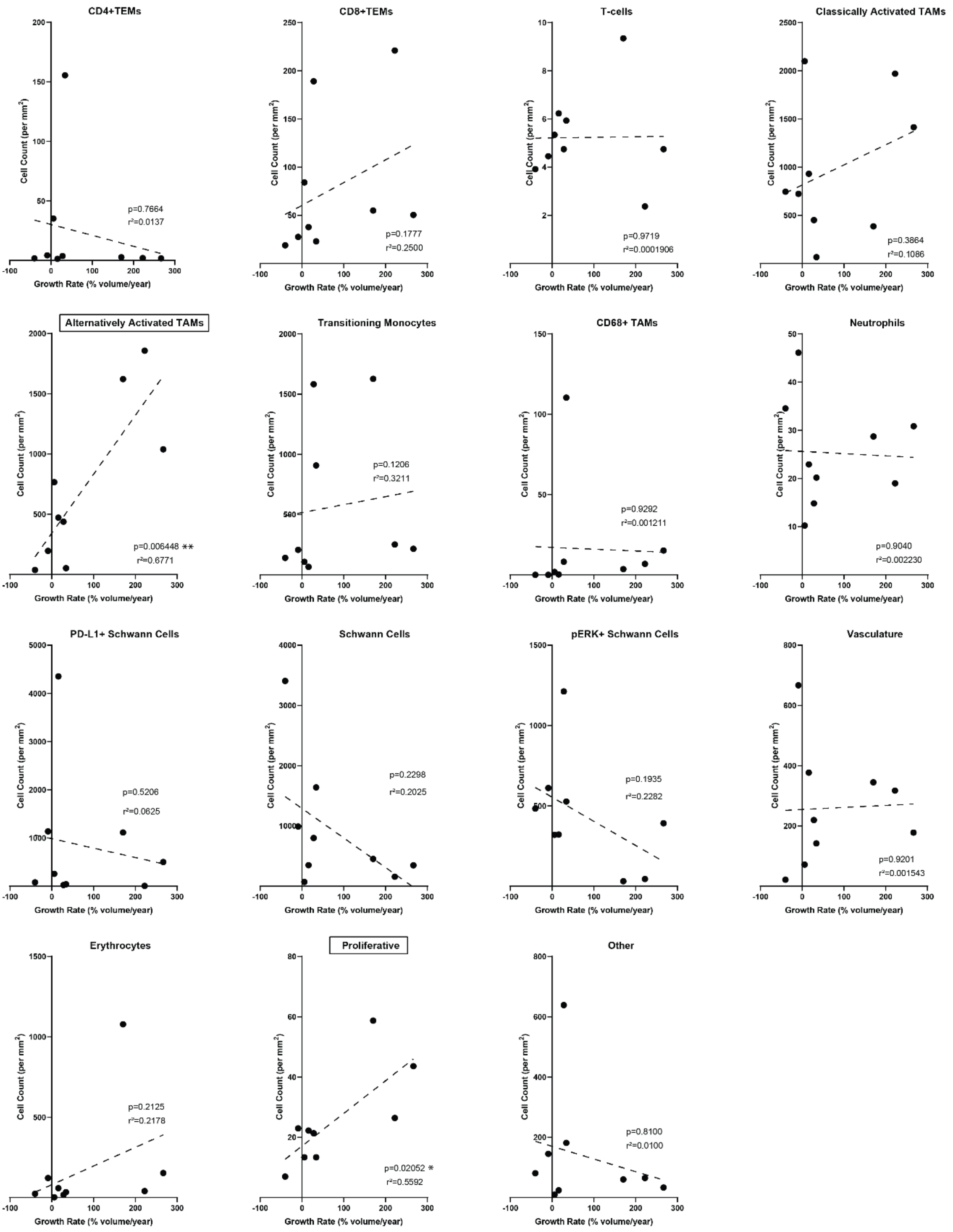

Supplementary Figure 3. Cell count per mm^2 of population cell types correlated against growth rate, where alternatively activated TAMs and proliferative cells significantly correlated with faster growing vestibular schwannoma. Vestibular schwannoma (VS) samples from static (N=4, 3 male, 1 female) and growing (N=5, 4 male, 1 female) patients. Static or growing VS were defined as volume change/year < 20% or ≥ 20%, respectively. Shapiro Wilk normality test followed by two tailed Pearson correlation or two tailed Spearman correlation with simple linear regression against growth rate. Correlation coefficient significance when $p < 0.05$.

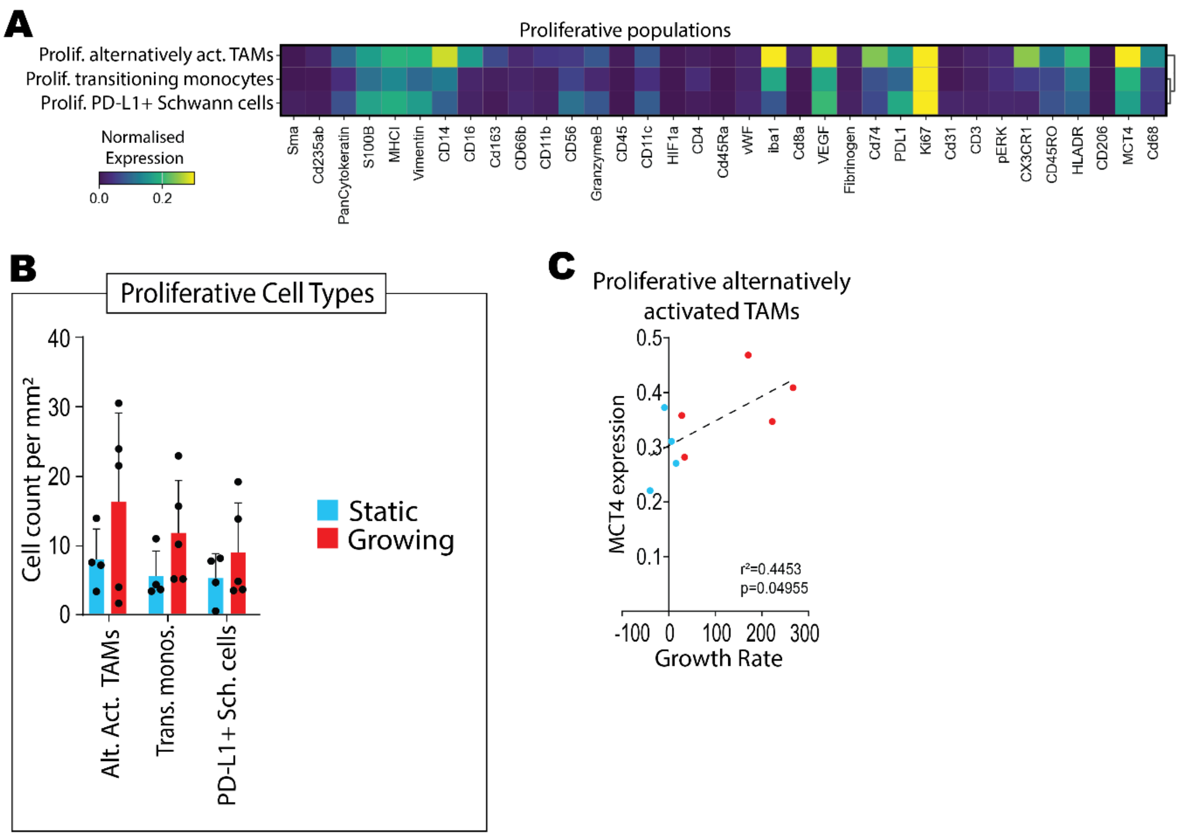

Supplementary Figure 4. Proliferative cell types within VS include alternatively activated TAMs, transitioning monocytes, and PD-L1+ Schwann cells. Vestibular schwannoma (VS) samples from 4 static (3 male, 1 female) and 5 growing (4 male, 1 female) patients. Static or growing VS were defined as volume change/year < 20% or ≥ 20%, respectively. **(A)** Heatmap expression of IMC panel markers as per Leiden sub-clustering of the proliferative group into three distinct proliferative cell types from all VS cases (N=9). **(B)** Proliferative cell type count per mm^2 per case for static (N=4) and growing (N=5) VS. Shapiro Wilk normality test followed by 2-way ANOVA, all not significant. **(C)** Of all IMC markers expressed by proliferative alternatively activated TAMs, only monocarboxylate transporter 4 (MCT4) significantly correlated with growth rate. Two tailed Pearson correlation with simple linear regression against growth rate. Statistical significance when $p < 0.05$. Abbreviations: proliferative (Prolif.), alternatively activated TAMs (Alt. Act. TAMs), transitioning monocytes (trans. monos), Schwann cells (Sch. cells).

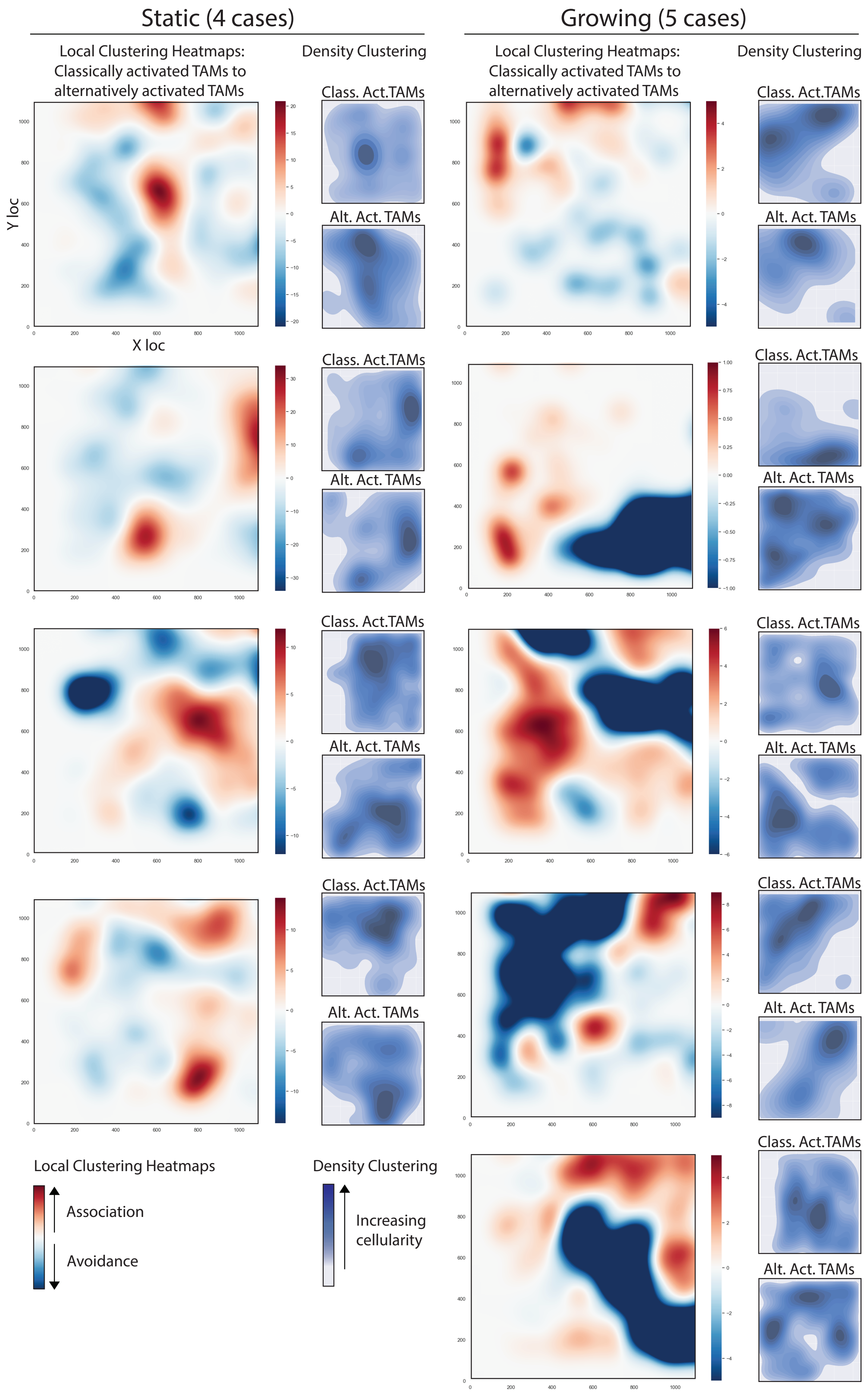

Supplementary Figure 5.Interaction and avoidance of classically activated and alternatively activated tumour associated macrophages (TAM) within vestibular schwannoma (VS) tissue. Local clustering heatmaps for positive and negative association of classically activated (Class. Act.) and alternatively activated (Alt. Act.) TAMs with matched density clustering plots for classically activated and alternatively activated TAM location within the VS tissue. VS samples from static (N=4, 3 male, 1 female) and growing (N=5, 4 male, 1 female) patients. Static or growing VS were defined as volume change/year < 20% or ≥ 20%, respectively.
